# Sorafenib targets and inhibits the oncogenic properties of endometrial cancer stem cells via the RAF/ERK pathway

**DOI:** 10.1186/s13287-022-02888-y

**Published:** 2022-06-03

**Authors:** Tomoka Takao, Hirotaka Masuda, Takashi Kajitani, Fumie Miki, Kaoru Miyazaki, Yushi Yoshimasa, Satomi Katakura, Shoko Tomisato, Sayaka Uchida, Hiroshi Uchida, Mamoru Tanaka, Tetsuo Maruyama

**Affiliations:** 1 Department of Obstetrics and Gynecology, Keio University School of Medicine, 35, Shinanomachi, Shinjukuku, Tokyo, 160-8582 Japan; 2 Department of Regenerative Science, Graduate School of Medicine, Dentistry and Pharmaceutical Sciences, Okayama University, 2-5-1, Shikatacho, Kitaku, Okayama, 700-8558 Japan; 3 Present Address: Sakura No Seibo Junior College, 3-6, Hanazonocho, Fukushima, 960-8585 Japan; 4 Present Address: Sho Hospital, 1-41-14, Itabashi, Tokyo, 173-0004 Japan

**Keywords:** Endometrial cancer, Cancer stem cells, Side-population, Sorafenib, HHUA

## Abstract

**Background:**

Distinct subsets of cancer stem cells (CSCs) drive the initiation and progression of malignant tumors via enhanced self-renewal and development of treatment/apoptosis resistance. Endometrial CSC-selective drugs have not been successfully developed because most endometrial cell lines do not contain a sufficient proportion of stable CSCs. Here, we aimed to identify endometrial CSC-containing cell lines and to search for endometrial CSC-selective drugs.

**Methods:**

We first assessed the presence of CSCs by identifying side populations (SPs) in several endometrial cancer cell lines. We then characterized cell viability, colony-formation, transwell invasion and xenotransplantion capability using the isolated SP cells. We also conducted real-time RT-PCR, immunoblot and immunofluorescence analyses of the cells’ expression of CSC-associated markers. Focusing on 14 putative CSC-selective drugs, we characterized their effects on the proliferation and apoptosis of endometrial cancer cell lines, examining cell viability and annexin V staining. We further examined the inhibitory effects of the selected drugs, focusing on proliferation, invasion, expression of CSC-associated markers and tumor formation.

**Results:**

We focused on HHUA cells, an endometrial cancer cell line derived from a well-differentiated endometrial adenocarcinoma. HHUA cells contained a sufficient proportion of stable CSCs with an SP phenotype (HHUA-SP). HHUA-SP showed greater proliferation, colony-formation, and invasive capabilities compared with the main population of HHUA cells (HHUA-MP). HHUA-SP generated larger tumors with higher expression of proliferation-related markers, Ki67, c-MYC and phosphorylated ERK compared with HHUA-MP when transplanted into immunodeficient mice. Among the 14 candidate drugs, sorafenib, an inhibitor of RAF pathways and multiple kinase receptors, inhibited cell proliferation and invasion in both HHUA-SP and -MP, but more profoundly in HHUA-SP. In vivo treatment with sorafenib for 4 weeks reduced the weights of HHUA-SP-derived tumors and decreased the expression of Ki67, ZEB1, and RAF1.

**Conclusions:**

Our results suggest that HHUA is a useful cell line for discovery and identification of endometrial CSC-selective drugs, and that sorafenib may be an effective anti-endometrial cancer drug targeting endometrial CSCs.

**Supplementary Information:**

The online version contains supplementary material available at 10.1186/s13287-022-02888-y.

## Background

Endometrial cancer is one of the most common gynecological malignancies in the industrialized world. The incidence of cancers of the corpus uteri, primarily of the endometrium, has been increasing continuously (a 1.3% increase per year from 2007 to 2016) [1, 2]. The most recent data revealed 65,620 estimated new cases and 12,590 estimated new deaths in 2020 in the U.S. [2]. Surgery and adjuvant chemotherapy, typically taxane- or platinum-based, are the main treatments for endometrial cancer. The majority of patients have early-stage disease at diagnosis and thus a good prognosis after surgery alone. However, even cytoreductive surgery together with adjuvant chemotherapy cannot effectively control advanced-stage or recurrent endometrial cancer, resulting in a lower than 20% survival rate [3]. Thus, a novel approach for the treatment of resistant, recurrent, and/or advanced endometrial cancer is required to improve outcomes.

Therapies targeting cancer stem cells (CSCs) and stemness-related pathways are one of the most intriguing strategies to overcome drug resistance, recurrence and metastasis [4]. CSCs are a subset of tumor cells with the ability to self-renew to generate the diverse cells that comprise the bulk of the tumor [4]. Therefore, CSCs contribute to tumor formation and enlargement via continuous production of numerous descendant cancer cells. In addition, they are generally resistant to exogenous insults such as anticancer drugs by pumping out the insulting agents via ATP-dependent efflux pumps, including multidrug resistance protein 1 (MDR1), thereby surviving even under unfavorable conditions [4]. Furthermore, the generation of CSCs occurs mainly via the activation of epithelial-to-mesenchymal transition (EMT) programs. Consequently, CSCs generally are highly motile with marked migratory and invasive properties [5, 6]. As a consequence, CSCs can easily detach from the original tumor, migrate, implant, and invade to a new site, ultimately leading to metastatic tumor formation. Thus, CSCs play critical roles in every aspect of cancer progression including tumor formation and enlargement, invasion, metastasis, and drug resistance, providing a rationale for targeting CSCs for cancer therapy [4–6]. Development of specific therapies targeting CSCs, therefore, holds hope for improving the survival and quality of life of cancer patients.

The presence of CSCs in endometrial cancer was first discovered in 2009 [7]. Since then, various types of endometrial CSCs have been identified and characterized using novel isolation procedures based on the expression of surface markers or biological activities, such as dye exclusion activity to isolate cells with the "side population (SP)" phenotype [8]; this has enabled elucidation of the potential roles of endometrial CSCs in endometrial cancer biology and etiology [8]. Despite the advances in endometrial CSC research, therapies targeting endometrial CSCs are lacking, partly because few endometrial cancer cell lines contain a sufficient proportion of stable CSCs.

Here, we aimed to identify a CSC-containing cell line among several endometrial cancer lines and to evaluate the effects of endometrial CSC-selective drugs on the identified cell line. We found that HHUA, an endometrial cancer cell line derived from well-differentiated endometrial adenocarcinoma [9], contained a sufficient proportion of stable CSCs among several endometrial cell lines tested. We then demonstrated that sorafenib is a promising endometrial CSC-selective anti-cancer drug among 14 candidate putative CSC-selective drugs evaluated.

## Methods

### Reagents

We assessed the following 14 putative CSC-targeting drugs as possible candidate endometrial CSC-targeting drugs: celecoxib [10] (Sigma-Aldrich, St. Louis, MO), genistein [11] (WAKO, Osaka, Japan), imatinib [12] (WAKO), itraconazole [13] (WAKO), lithium carbonate [14] (Nacalai Tesque, Kyoto, Japan), niclosamide [10, 12] (Santa Cruz Biotechnology, Dallas, Tx), retinyl acetate [15] (Sigma-Aldrich,), bevacizumab [16] (Roche, Basel, Switzerland), everolimus [16] (WAKO), ramucirumab [16] (Eli Lilly, Indianapolis, Indiana), sorafenib [16] (LC Laboratories, Woburn, MA), temsirolimus [16, 17] (WAKO), thalidomide [16] (LKT Laboratories, Inc., MN), and tranilast [17] (Tokyo Chemical Industry co., Ltd., Japan). The 14 drugs can be categorized into three groups based on their pharmacological actions: Wnt/β-catenin inhibitors (celecoxib, genistein, imatinib, itraconazole, lithium carbonate, niclosamide and retinyl acetate), angiogenesis inhibitors (bevacizumab, everolimus, ramucirumab, sorafenib, temsirolimus and thalidomide), and EMT inhibitors (tranilast). Reagent concentrations for assays were as follows: imatinib, 10 µM; niclosamide, 0.5 µM; sorafenib, 10 µM; genistein, 40 µM; tranilast, 50 µM; itraconazole, 10 µM; celecoxib, 100 µM; lithium carbonate, 40 µM; retinyl acetate, 40 µM; temsirolimus, 20 µM; thalidomide, 1 µM; bevacizumab, 1 µM; ramucirumab, 1 µM; and everolimus, 0.2 µM.

### Cell culture

The HHUA human endometrial cancer cell line [9] was obtained from the RIKEN BioResource Center (Ibaraki, Japan). HHUA cells were maintained as monolayers at 37 °C in 5% CO_2_/air in Dulbecco’s modified Eagle’s Medium/Ham’s F12 (DMEM/F12, WAKO, Japan) supplemented with 10% heat-inactivated fetal bovine serum (FBS) and antibiotic/antimycotic solution (Sigma-Aldrich). HEC1 and HEC6 cells were obtained from the Health Science Research Resources Bank (Osaka, Japan). THESC cells, an endometrial stromal cell line, was purchased from ATCC® (CRL-4003TM). Ishikawa cells (clone 3-H-12) [18], a well-differentiated human endometrial adenocarcinoma cell line, were given by Dr. Masato Nishida. hEM cells, an immortalized human endometrial glandular cell line, were a kind gift from Dr. Satoru Kyo [19].

### Isolation of SP cells

HHUA cells were detached from the culture dishes using trypsin and EDTA, washed, and suspended at a concentration of 2 × 10^6^/mL in culture medium. Then, 7.5 mg/mL Hoechst 33342 (Molecular Probes, Eugene, OR) was added to the cells, either alone or in combination with reserpine (Sigma-Aldrich), for 90 min at 37 °C. Finally, the cells were counterstained with 1 µg/mL propidium iodide to label dead cells. Flow cytometric analysis and cell sorting were performed on a triple laser MoFlo (Cytomation, Fort Collins, CO) with Summit software (Cytomation) or a FACSVantage SE flow cytometer (BD Biosciences, San Jose, CA) with CELLQuest (BD Biosciences) software. Hoechst 33342 was excited at 350 nm, and the fluorescence emission was detected using 405/BP (band pass) 30 and 570/BP20 optical filters for Hoechst blue and Hoechst red, respectively, and a 550 nm long-pass dichroic mirror (Omega Optical Inc., Brattleboro, VT) to separate the emission wavelengths. Both Hoechst blue and red fluorescence intensities are shown on a linear scale. Propidium iodide fluorescence was measured through 630/BP30 after excitation at 488 nm with an argon laser, and a live cell gate excluded propidium iodide-positive cells.

### Cell viability assay

HHUA cells were seeded into 96-well plates at 5 × 10^3^ cells/well with or without drugs and maintained for 5 days. Cellular proliferation/survival was quantitatively determined using 3-(4,5-dimethylthiazol-2-yl)-5-(3-carboxymethoxyphenyl)-2-(4-sulfophenyl)-2H-tetrazolium, inner salt (MTS), a reagent that is reactive with metabolically active (viable) cells. We used the Cell Titer 96® AQueous One Solution Cell Proliferation Assay kit (Promega, Madison, Wisconsin) and measured absorbance on days 1, 3 and 5. Twenty µL of MTS solution was added to 100 µL of medium and incubated for 1 h at 37 °C, followed by spectrophotometric quantification at 490 nm.

### Self-renewal assay

Cells were plated in 60 mm dishes at 100/cm^2^. Colonies were monitored to ensure that they were derived from single cells. The cloning dishes were stained with Giemsa solution (Merck, Germany).

### Invasion assay

Transwell inserts (Corning, NY) equipped with polycarbonate filters with 8 µm pores were used. The upper surface of the filters was coated with 2 mg/mL Matrigel® Matrix (Corning). A total of 2 × 10^5^ cells were suspended in 200 µL DMEM/F12 and plated on Matrigel. Medium supplemented with 10% FBS was added to the lower well. After 24 h of culture, the upper insert was washed with PBS and scraped with cotton swabs. The invading cells on the bottom surface of the filter were fixed with methanol, stained with Giemsa, and counted under the BIOREVO BZ-9000 fluorescence microscope (Keyence, Osaka, Japan).

### Annexin V staining and flow cytometry

HHUA cells were treated with or without drugs for 2 and 7 days and then harvested by trypsinization and washed twice with cold PBS. The cells were centrifuged at 1500 rpm for 5 min, and then the supernatant was discarded. The cell pellet was resuspended in HBSS + buffer at a density of 1.0 × 10^5^ cells/100 μL. The sample solution (100 μL) was incubated with 5 μL FITC-conjugated Annexin V (BioLegend, San Diego, CA) and 5 μL propidium iodide for 15 min at room temperature in the dark. HBSS + buffer (400 μL) was added to each sample tube, and the samples were subjected to flow cytometry using the Gallous flow cytometer (Beckman Coulter). The data were analyzed with flow cytometry analysis software Kaluza (Beckman Coulter) or FlowJo v10.7.1 software.

### Real-time reverse transcription-polymerase chain reaction

RNA was extracted from cells using the ReliaPrep RNA cell miniprep system (Promega). Reverse transcription was performed using PrimeScript™ RT Reagent Kit (TAKARA, Japan). qRT-PCR was performed using SYBR® Premix Ex Taq™ II (TAKARA). The expression level of *ACTB* was used as an internal control for mRNA expression. Gene expression levels were quantified using the ΔΔCt (where Ct is the threshold cycle) method. qRT-PCR was performed in three independent samples to confirm reproducibility. The following primers were used: *MDR1* forward, 5′-TGGTTTGATGTGCACGATGT-3′, and reverse, 5′-GGCCAAAATCACAAGGGTTA-3′; *ABCG2* forward, 5′-GGCCTCAGGAAHACTTATGT-3′, and reverse, 5′-AAGGAGGTGGTGTAGCTGAT-3′; *CTNNB1* forward, 5′-GCTTTCAGTTGAGCTGACCA-3′, and reverse, 5′- CAAGTCCAAGATCAGCAGTCTC-3′; and, *ACTB* forward, 5′-AGAAAATCTGGCACCACACC-3′, and reverse, 5′- AGAGGCGTACAGGGATAGCA -3′.

### Protein extraction and immunoblot analysis

HHUA cells and tumors were harvested using M-PER Mammalian Protein Extraction Reagent (Thermo Fisher Scientific) supplemented with protease inhibitor (Roche, Germany) and phosphatase inhibitor (WAKO). Proteins (40 μg) were separated by 4–20% SDS–polyacrylamide gel electrophoresis, followed by transfer to polyacrylamide membranes and blocking using Blocking One reagent (Nacalai Tesque) for 1 h at room temperature. Then, the membranes were incubated overnight at 4 °C with primary antibodies against RAF1, pERK, ERK, p-AKT, AKT (all from CST, Danvers, MA), c-MYC, cyclin D2, ZEB1, and α-tubulin (all from Santa Cruz Biotechnology). The primary antibodies were detected using a 1:1000 dilution of horseradish peroxidase-conjugated goat anti-rabbit IgG or anti-mouse IgG secondary antibodies (Santa Cruz Biotechnology). All membranes were visualized using ImmunoStar LD chemiluminescence reagent (WAKO). Images were captured using a charge-coupled device camera (LAS4000 mini; FujiFilm, Tokyo, Japan), and the bands were quantified using ImageJ software.

### Mice and HHUA cell transplantation

Four- to five-week-old female ICR null/null (nude) mice were obtained from Charles River Laboratories Japan, Inc. (Yokohama, Japan). All mice were maintained under specific-pathogen-free conditions in accordance with the guidelines for the Care and Use of Laboratory Animals of the Keio University School of Medicine. All mouse experiments were performed under approval of the Institutional Animal Care and Use Committee of Keio University School of Medicine (approval numbers 16066–1, 160660–2, and 17037–3). HHUA cells (1 × 10^5^) were suspended in 100 μL BD Matrigel Basement Membrane Matrix (BD Biosciences, Bedford, MA) and injected subcutaneously into both hind legs of the nude mice (n = 25). Six weeks after injection, the mice were treated orally with sorafenib at 30 mg/kg/day for 2 or 4 weeks and then sacrificed, followed by tumor excision.

### Histological and immunofluorescence analysis

Graft-bearing tumors excised from nude mice were embedded in Tissue-Tek OCT compound (Sakura Finetek, Torrance, CA), frozen, and serially sectioned at 5 µm by using a Leica cryostat (Leica Microsystems Inc.). Some samples were fixed in 4% PFA for 10 min. at room temperature and permeabilized using 0.1% Triton X-100 in PBS for 5 min. After blocking with 10% bovine serum albumin for 40 min, tissue sections were incubated with one of the following: primary antibodies against MDR1 or RAF1 (both from CST), vimentin (VM) (DAKO, Santa Clara, CA), beta-catenin/CTNNB1 (Chemicon International, USA), BCL2 (Santa Cruz Biotechnology), ZEB1 (Sigma-Aldrich) or Ki67 (Santa Cruz Biotechnology). After overnight treatment at 4 °C, sections were exposed to a secondary antibody: Alexa Fluor 546-labeled goat anti-rabbit IgG (A11035), Alexa Fluor 546-labeled goat anti-mouse IgG (A11030), or Alexa Fluor 488-labeled goat-anti-rabbit (A11043) (Invitrogen/Life Technologies) for 60 min at room temperature. Nuclei were counterstained with mounting medium containing DAPI (Thermo Fisher Scientific). Cells were visualized under the BIOREVO BZ-9000 fluorescence microscope (Keyence, Osaka, Japan).

### Statistical analyses

The data are presented as the mean ± SEM of three independent experiments. Differences between the test and control conditions in this study were analyzed using Student’s *t*-test. All statistical analyses were performed using SPSS 17.0 statistical software. A *P*-value < 0.05 was considered statistically significant.

## Results

### HHUA cells contained an SP cell fraction that harbored CSC-like properties

**Fig. 1:**
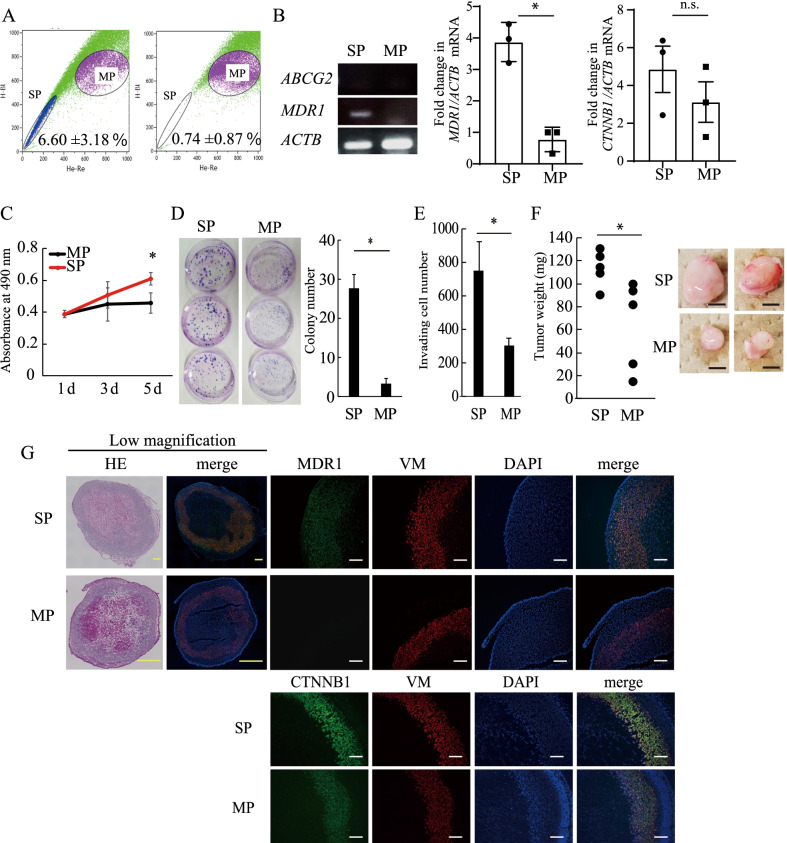
Isolation and characterization of CSC-like cells from the human endometrial cancer cell line HHUA. **A** Flow cytometric determination of the distributions of the side population (SP) and main population (MP) in living HHUA cells stained with Hoechst 33342 in the absence (left) or presence (right) of reserpine. Treatment with 50 μM reserpine resulted in the disappearance of the SP fraction. **B** Expression of ABCG2, MDR1 and ACTB mRNAs in HHUA-SP and -MP cells as determined by qRT-PCR. Expression of MDR1 and CTNNB1 mRNAs in HHUA-SP and -MP cells as determined by real-time RT-PCR. **C** Proliferation of HHUA-SP and -MP cells as determined by MTS assay. Each point indicates the mean ± SEM absorbance at 490 nm obtained from three independent experiments using three individual samples. *, *P* < 0.05, vs. DMSO control, based on Student’s *t*-test. **D** Colony formation ability of HHUA-SP and -MP cells. HHUA-SP and -MP cells were cultured in the cloning plates as indicated and then stained with Giemsa solution. The bar graph shows the mean ± SEM colony number obtained from three independent experiments. *, *P* < 0.05, based on Student’s *t*-test. **E** Cell invasion ability of HHUA-SP and -MP cells as determined by cell invasion assay. Each bar indicates the mean ± SEM number of invading cells obtained from three independent experiments using three individual samples, *, *P* < 0.05, based on Student’s *t*-test. **F** Weight and gross appearance of tumors derived from HHUA-SP and -MP cells at 6 weeks after inoculation into the subcutaneous tissue of nude mice. Each dot indicates the tumor weight of an individual mouse. *, *P* < 0.05, based on Student’s *t*-test. Scale bars, 1 mm. **G** Hematoxylin and eosin and immunofluorescence staining of HHUA-SP or -MP-derived tumors using antibodies against vimentin (VM) and MDR1 or CTNNB1. DAPI was used for nuclear staining. Scale bars, 1 mm (yellow) and 200 μm (white)

CSCs are defined as a subset of tumor cells capable of self-renewal and generation of diverse cells that comprise the bulk of the tumor [20]. The two abilities can be determined by colony formation and xenotransplantation assays, respectively. We determined the presence of the SP fraction in various cell lines derived from endometrial cancers or normal endometria. Thus, Ishikawa, HEC1, HEC6, HHUA, THESC and hEM cells were incubated with Hoechst 33342 in the presence or absence of the MDR inhibitor reserpine and subjected to flow cytometry analysis (Additional file 1: Fig. S1). Among these cell lines tested, we found that only HHUA endometrial cancer cells stably and consistently contained a reserpine-sensitive SP fraction (HHUA-SP) that comprised 6.60 ± 3.18% (mean ± SEM) of the total cell population (Fig. 1A, left). The HHUA-SP fraction was greatly reduced following treatment with reserpine (Fig. 1A, right). MDR1, but not ABCG2, mRNA expression was more prominent in HHUA-SP than in the main population of HHUA cells (HHUA-MP), as determined by RT-PCR and real-time qPCR (Fig. 1B). In addition, as compared to HHUA-MP, HHUA-SP expressed more transcripts of *CTNNB1*, a difference that did not reach significance (Fig. 1B). *CTNNB1* is a marker for self-renewal ability in various types of adult stem cells and CSCs including endometrial CSCs [21]. Cell growth assays conducted for 5 days revealed that HHUA-SP cells proliferated faster than HHUA-MP cells (Fig. 1C). Furthermore, to test the self-renewal capability of HHUA-SP and -MP cells, they were plated in 60 mm dishes (100/cm^2^) and allowed to proliferate and form colonies. HHUA-SP cells produced large densely packed colonies, whereas HHUA-MP cells generated small, low-density colonies (Fig. 1D, left panels). The number of large (> 2 mm) colonies was greater among HHUA-SP than HHUA-MP cells (27.6 ± 3.40 vs. 3.33 ± 1.25) (Fig. 1D, right panel). Cell invasion assays showed that HHUA-SP cells were more invasive than HHUA-MP cells (Fig. 1E).

To examine the ability of HHUA-SP and -MP cells to form tumors in vivo, we transplanted HHUA-SP and -MP cells into nude mice and characterized the resulting tumors at 6 weeks after transplantation. As shown in Fig. 1F, HHUA-SP-tumors derived were heavier and larger than HHUA-MP-derived tumors. Both tumor types were positive for human VM, indicating a human cell origin (Fig. 1G). Notably, MDR1 was strongly expressed in HHUA-SP-derived tumors, whereas MDR1 expression was absent in HHUA-MP-derived tumors (Fig. 1G), which suggests that MDR1-positive HHUA-SP cell expansion contributed, at least in part, to HHUA-SP-derived tumor formation. Also, HHUA-SP-derived tumors expressed more CTNNB1 as compared to HHUA-MP-derived tumors (Fig. 1G), suggesting that enhanced tumor formation by HHUA-SP might be attributable to a higher self-renewal capacity of HHUA-SP. Thus, HHUA cells contain a SP that exhibits CSC-like properties, including high capacities for colony formation, cell proliferation, cell invasion, and in vivo tumor formation, indicating that this cell line is appropriate for evaluating endometrial CSC-targeting drugs.

### Sorafenib inhibited proliferation, induced apoptosis and reduced the size of the SP fraction among HHUA cells

**Fig. 2:**
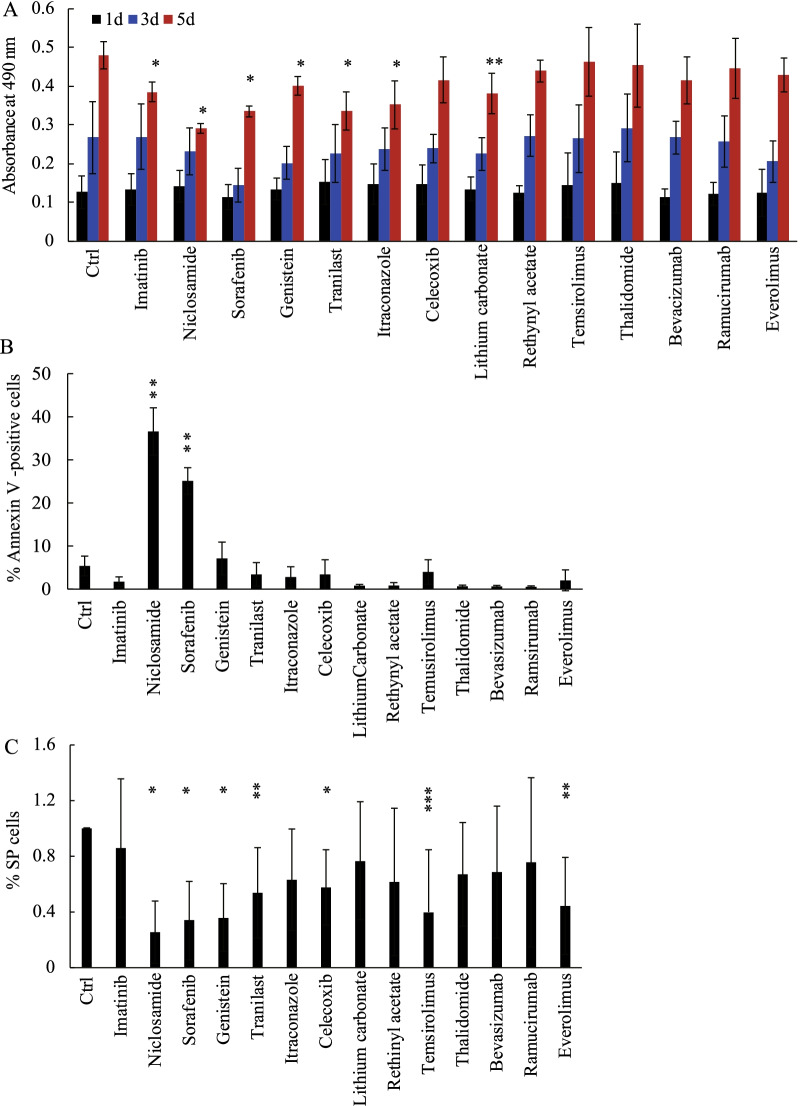
Effects of putative stem cell-selective candidate drugs on proliferation and apoptosis in HHUA cells. **A**–**C** Effects of the candidate drugs on HHUA cell proliferation as determined by MTS assay (**A**), HHUA cell apoptosis was determined by annexin V-staining (**B**), and the percentage of HHUA-SP cells among all HHUA cells was determined by flow cytometric analysis. **C** Each bar indicates the mean ± SEM absorbance at 490 nm (**A**), the percentage of annexin V-positive cells (**B**), and the percentage of HHUA-SP cells among all HHUA cells **C** obtained from three independent experiments using three individual samples. *, *P* < 0.01, **, *P* < 0.02, and ***P < 0.05, versus DMSO control (Ctrl), based on Student’s *t*-test

We investigated the effects of the 14 candidate drugs specified in the Materials and Methods section on the growth of HHUA cells using the MTS assay. We aimed to identify drugs that inhibit the properties and behavior of endometrial cancer cells, particularly endometrial CSCs, without affecting normal endometrial cells. Thus, we determined the concentration of each drug at which its inhibitory effect on the growth of the normal human endometrial cell line THESC was less than 50% compared with the vehicle control (data not shown). Most of the drugs significantly inhibited HHUA cell growth (Fig. 2A).

To examine the effect of these candidate drugs on apoptosis, HHUA cells were treated with each drug for 2 and 7 days and subjected to annexin V staining followed by flow cytometry for identification of total apoptotic cells (i.e., annexin V-positive cells). No apparent apoptosis-inducing effects were observed for any of the drugs used to treat HHUA cells after 2 days of exposure (data not shown), but after 7 days of treatment, sorafenib and niclosamide significantly induced apoptosis compared with the vehicle control (Fig. 2B and Additional file 2: Fig. S2). We then analyzed the proportion of SP cells among all HHUA cells after 2 days of treatment with each drug. Several drugs, including sorafenib and niclosamide, significantly decreased the proportion of HHUA-SP cells (Fig. 2C). It is possible that the decrease in the SP proportion mediated by sorafenib on day 2 might have been due to arrested cell growth and/or cell death. Given that HHUA-SP cells constituted only about 6% of the entire HHUA population, the behavior of HHUA-SP cells might have been masked by that of HHUA-MP cells (94% of the culture). The two-day treatment with sorafenib might have damaged the SP cell fraction, resulting in fragile and aberrant SP cells. Because SP cells act as CSCs and thereby give rise to MP cells, such damage to SP cells might have led to apoptotic death of a significant fraction of the entire HHUA population after 7 days. Thus, endometrial CSC-targeting drugs are expected to inhibit CSCs, the minor but key cancer-initiating/forming cells and thereby to reduce resultant non-CSCs in the majority of cancer cells, ultimately resulting in the inhibition of the tumor formation.

Taken together, these results suggested that sorafenib and niclosamide are the best endometrial CSC-targeting drugs in terms of CSC growth inhibition and apoptosis induction, prompting us to further verify the validity of these drugs. Niclosamide is an anthelmintic drug that has been used for over 50 years mainly to treat tapeworm infections [22], and it has recently shown great promise in treating many diseases including cancer [22]; however, it is not currently available in the U.S. or Japan. Because sorafenib is widely used, mainly for treatment of hepatocellular carcinoma and renal cell carcinoma, we chose sorafenib for further analyses.

### Sorafenib inhibited the proliferation and invasion of HHUA-SP and -MP cells

**Fig. 3:**
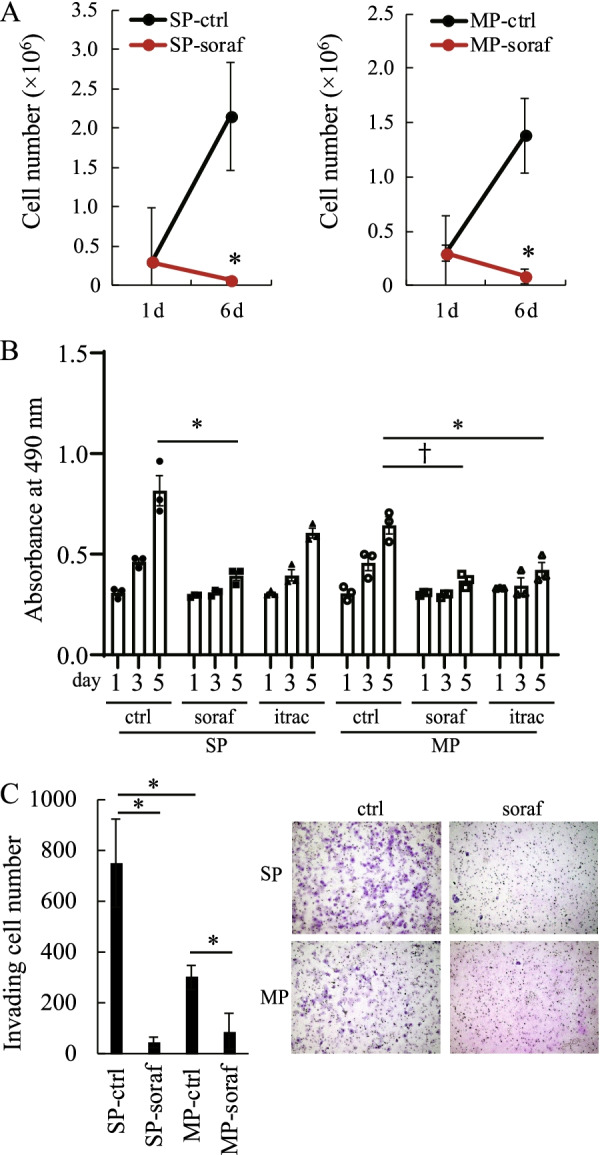
Sorafenib inhibition of HHUA cell properties and stem cell characteristics. **A** Reduction of HHUA-SP and -MP cell numbers by treatment with sorafenib (soraf), as determined using an automated cell counter. Each dot indicates the mean ± SEM obtained from three independent experiments using three individual samples. *, *P* < 0.05 vs. DMSO control (ctrl), based on Student’s *t*-test. **B** The effects of soraf and itraconazole (itrac) on HHUA-SP and -MP cell proliferation, as determined by the MTS assay. Each point indicates the mean ± SEM absorbance at 490 nm obtained from three independent experiments using three individual samples. *, *P* < 0.01, †, *P* < 0.02 versus each control, based on Student’s *t*-test. **C** Inhibition of HHUA-SP and -MP cell invasion by soraf. Each bar indicates the mean ± SEM number of invading cells obtained from three independent experiments using three individual samples. Images (insert) show representative Giemsa-stained membranes followed by treatment as indicated. *, *P* < 0.05 vs. DMSO control, based on Student’s *t*-test

To examine the growth inhibitory effect of sorafenib on endometrial CSCs, we isolated HHUA-SP or -MP cells from the whole HHUA cell population and subjected each to cell proliferation assays. Treatment with sorafenib inhibited both HHUA-SP and -MP cell growth (Fig. 3A, left and right panels, respectively). The numbers of both HHUA-SP and -MP cells decreased after 6 days of treatment with sorafenib (Fig. 3A), suggesting that sorafenib might induce apoptosis (Fig. 2B) and thereby decrease the number of HHUA cells. To evaluate the growth inhibitory effect and CSC selectivity of sorafenib, we compared the growth inhibitory effects of sorafenib and itoraconazole on HHUA-SP and -MP cells. We observed that itoraconazole inhibited HHUA cell growth (Fig. 2A) but did not affect the proportion of HHUA-SP cells (Fig. 2C). Sorafenib inhibited the proliferation of both HHUA-SP and -MP cells, whereas itoraconazole inhibited the proliferation of HHUA-MP cells, but not HHUA-SP cells (Fig. 3B). These results indicate that sorafenib, but not itoraconazole, has potential as a CSC-targeting drug, although both drugs inhibited the growth of the whole population of HHUA cells (Fig. 2A). We then tested whether sorafenib affects the invasion of HHUA-SP and -MP cells. A Transwell invasion assay revealed that sorafenib inhibited the invasion of both HHUA-SP and -MP cells (Fig. 3C). HHUA-SP cells exhibited higher proliferative and invasive potentials than did HHUA-MP cells (Figs. 1C, E and 3A, B, C). Treatment with sorafenib reduced the potentials of HHUA-SP and HHUA-MP cells to comparable levels (Fig. 3A–C), suggesting that the inhibitory effects of sorafenib on HHUA-SP cells were more profound and selective than those on HHUA-MP cells. Thus, sorafenib inhibited the proliferation and invasion of both HHUA-SP and HHUA-MP cells but more profoundly and selectively affected HHUA-SP.

### Sorafenib treatment for 4 weeks suppressed tumor formation by HHUA-SP cells and MDR1 and Ki67 expression in HHUA-SP-derived tumors

**Fig. 4:**
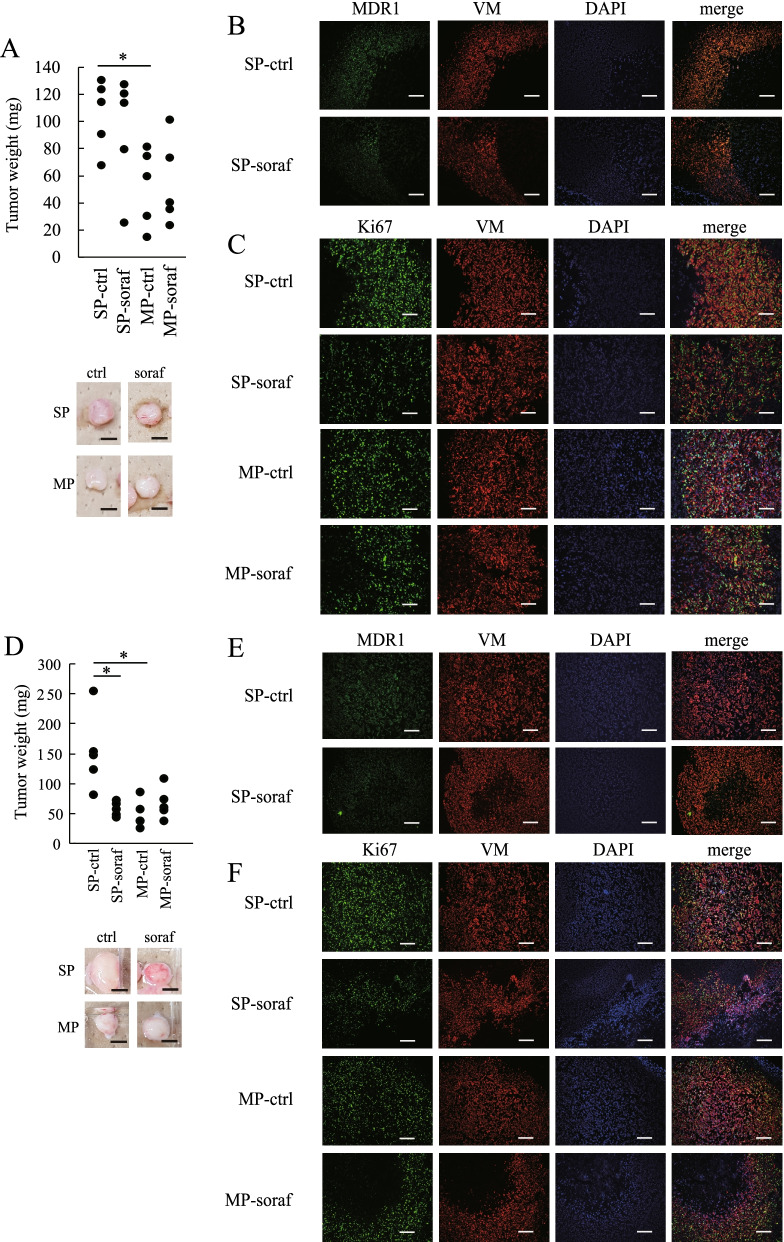
Inhibition of in vivo tumor formation from HHUA-SP cells by long-term treatment with sorafenib. **A** Weight of tumors derived from HHUA-SP or -MP cells 2 weeks after oral treatment with or without sorafenib (soraf and ctrl, respectively). Before treatment, tumors formed 6 weeks after inoculation of HHUA-SP or -MP cells into the subcutaneous tissue of nude mice. Each dot indicates the tumor weight of an individual mouse. Images show representative tumors. *, *P* < 0.05, based on Student’s *t*-test. Scale bars, 1 mm. **B** Immunofluorescence staining of HHUA-SP-derived tumors treated as in **A** for 2 weeks using antibodies against VM and MDR1. DAPI was used for nuclear staining. Scale bars, 200 μm. **C** Immunofluorescence staining of HHUA-SP- and -MP-derived tumors treated as in **A** for 2 weeks using antibodies against VM and Ki67. DAPI was used for nuclear staining. Scale bars, 200 μm. **D** Weight or volume of tumors derived from HHUA-SP or -MP cells 4 weeks after treatment with or without sorafenib (soraf and ctrl, respectively). Tumor formation was assessed 6 weeks after inoculation of HHUA-SP or -MP cells into the subcutaneous tissue of nude mice. Each dot indicates the tumor weight of an individual mouse. Images show representative tumors. *, *P* < 0.05, based on Student’s *t*-test. Scale bars, 1 mm. **E** Immunofluorescence staining of HHUA-SP-derived tumors treated as in **D** for 4 weeks using antibodies against vimentin (VM) and MDR1. DAPI was used for nuclear staining. Scale bars, 200 μm. **F** Immunofluorescence staining of HHUA-SP- and -MP-derived tumors treated as in **D** for 4 weeks using antibodies against vimentin (VM) and Ki67. DAPI was used for nuclear staining. Scale bars, 200 μm

We investigated the effect of sorafenib on the in vivo formation of tumors derived from HHUA-SP and HHUA-MP cells. HHUA-SP or -MP cells were subcutaneously transplanted into nude mice and allowed to form tumors for 4 weeks, after which the mice were orally administered sorafenib or the vehicle control (DMSO) for 2 weeks. Tumor weight was significantly higher among HHUA-SP- than HHUA-MP-derived tumors (Fig. 4A), which is consistent with the data shown in Fig. 1F. Two weeks of treatment with sorafenib, however, did not affect either HHUA-SP or—MP-derived tumor weight compared with DMSO treatment. The MDR1 staining intensity in HHUA-SP-derived tumors was weaker after treatment with sorafenib than DMSO (Fig. 4B and Additional file 3: Fig. S3A). There were no apparent differences in the staining intensities of Ki67 or VM between HHUA-SP and -MP-derived tumors or between treatment with or without sorafenib, although Ki67 expression appeared to be suppressed in HHUA-SP- and MP-derived tumors upon sorafenib treatment. (Fig. 4C and Additional file 3: Fig. S3B). In contrast to 2 weeks of treatment, 4 weeks of sorafenib significantly reduced tumor weight and volume (Fig. 4D and Additional file 4: Fig. S4A), together with decreased staining intensity of MDR1 (Fig. 4E and Additional file 4: Fig. S4B) and Ki67 (Fig. 4F and Additional file 4: Fig. S4C), in HHUA-SP-derived tumors. In addition, we examined the expression of BCL2, an anti-apoptotic protein, in SP-derived and MP-derived tumors in mice treated with or without sorafenib for 4 weeks. The expression of BCL2 was decreased upon sorafenib treatment in HHUA-MP-derived tumors, whereas there was no apparent difference in the expression levels of BCL2 between control and sorafenib treatment in HHUA-SP-derived tumors (Additional file 5: Fig. S5). These results suggest that the apoptosis-inducing effect of sorafenib acted mainly on HHUA-MP cells rather than HHUA-SP cells.

### Sorafenib suppressed tumorigenesis-associated signalingpathways in vitro and in vivo

**Fig. 5:**
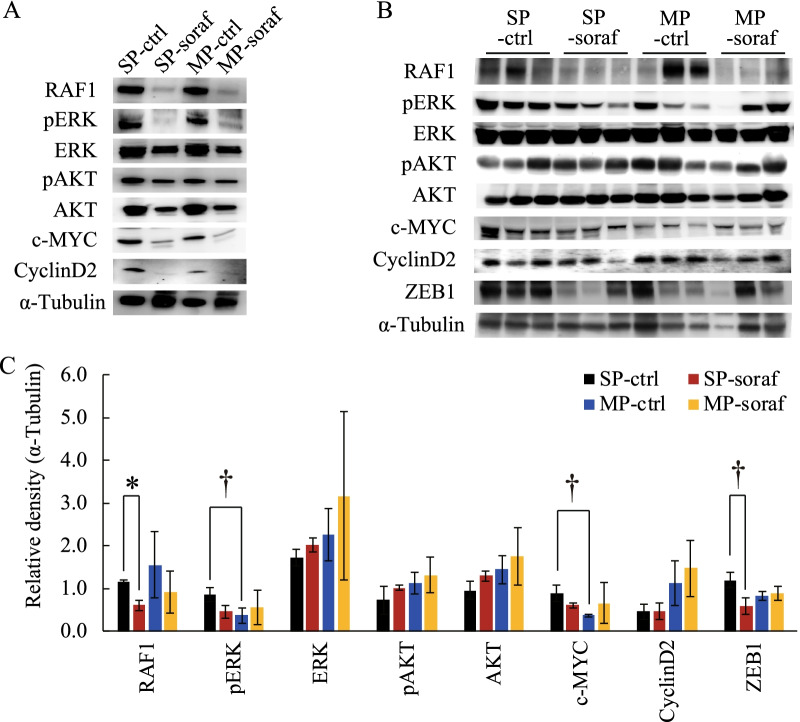
Effects of sorafenib on tumorigenesis-associated signaling pathways in vitro and in vivo. **A** Immunoblot analyses of RAF1, pERK, ERK, pAKT, AKT, c-MYC, cyclin D2, and α-tubulin in cultured HHUA-SP and -MP cells treated with or without sorafenib for 5 days. **B** Immunoblot analyses of RAF1, pERK, ERK, pAKT, AKT, c-MYC, cyclin D2, ZEB1, and α-tubulin in HHUA-SP- and -MP-derived tumors in mice treated orally with or without sorafenib for 4 weeks. **C** Each bar indicates the mean ± SEM relative density of each indicated protein obtained from three individual HHUA-SP- and -MP-derived tumor samples, as described in (**B**). The protein levels were normalized to that of α-tubulin. *, *P* < 0.01; †, *P* < 0.05, based on Student’s *t*-test

To address the molecular mechanisms underlying sorafenib-induced inhibition of cell proliferation, cell invasion, and in vivo tumor formation, we first examined the effect of sorafenib on the expression of tumorigenesis-associated signaling proteins (RAF1, ERK, c-MYC, cyclin D2, and AKT) in cultured HHUA-SP and -MP cells. RAF1 is a representative target protein of sorafenib, and the downstream effectors of RAF1 include ERK, c-MYC, and cyclin D2; AKT is a sorafenib-sensitive kinase. Immunoblot analyses revealed that RAF1, ERK, c-MYC, cyclin D2, and AKT were all downregulated or dephosphorylated (inactivated) in both cultured HHUA-SP and -MP cells after treatment with sorafenib (Fig. 5A). We next investigated the expression and phosphorylation status of the aforementioned tumorigenesis-associated signaling proteins, plus ZEB1, an EMT-related transcription factor that promotes cancer progression [23]. We focused on HHUA-SP- and -MP-derived tumors in mice that had been orally treated with or without sorafenib for 4 weeks (Fig. 5B). Densitometric analysis of immunoblots revealed higher levels of pERK and c-MYC in HHUA-SP- than HHUA-MP-derived tumors, and lower levels of RAF1 and ZEB1 in sorafenib-treated than vehicle-treated HHUA-SP-derived tumors (Fig. 5C). The pERK/ERK ratio was significantly lower in SP-soraf than in SP-ctrl, indicating that sorafenib acted as a kinase inhibitor and thereby effectively reduced the phosphorylation status of ERK (Additional file 6: Fig. S6). Similar to the pERK/α-tubulin ratio (Fig. 5C), the pERK/ERK ratio was higher in SP-ctrl than in MP-ctrl, but the difference was not quite significant (Additional file 6: Fig. S6, *P* = 0.068). Because ERK is known to be overexpressed in some tumor cells [24], it is possible that the α-tubulin may be more stable and appropriate as the internal control. In contrast to pERK/ERK, pAKT/AKT ratios were almost constant regardless of the presence or absence of sorafenib (Additional file 6: Fig. S6).

**Fig. 6:**
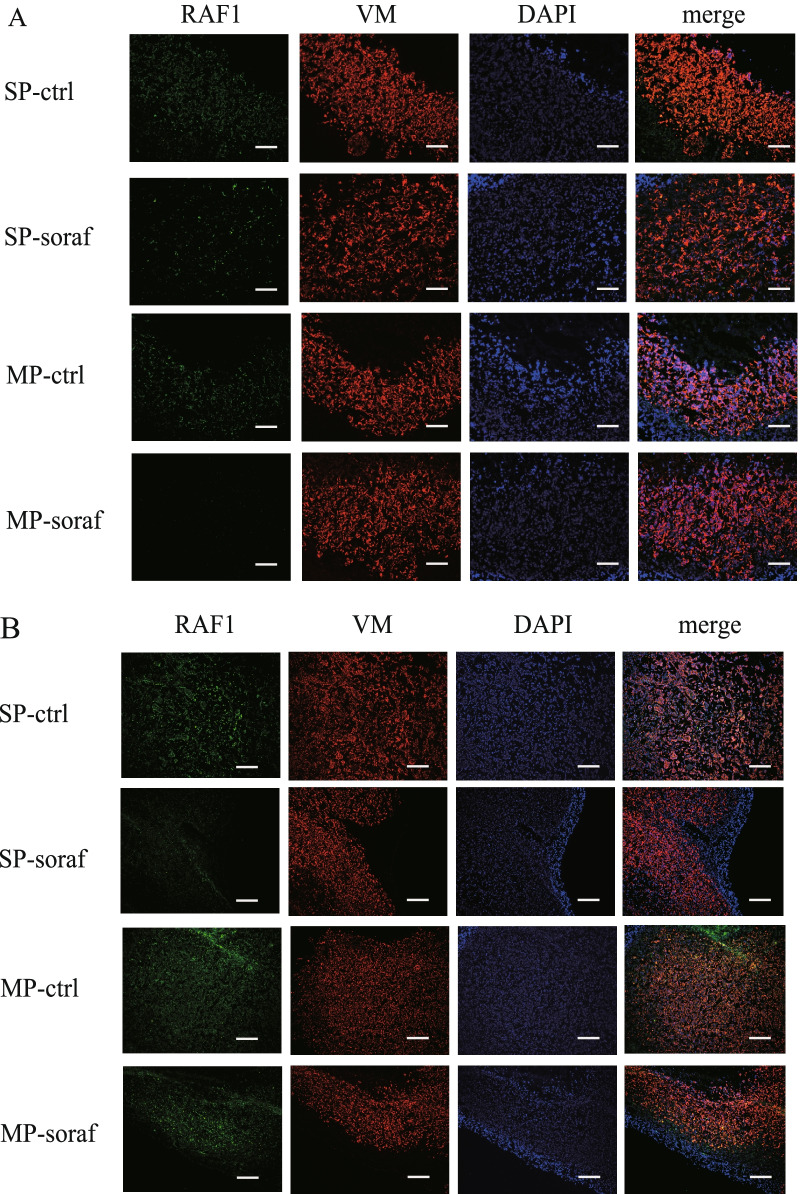
Effects of sorafenib on RAF1 expression in HHUA-SP- and -MP-derived tumors in mice. Immunofluorescence staining of RAF1 and VM in HHUA-SP- and -MP-derived tumors in mice treated orally with or without sorafenib for 2 weeks (**A**) or 4 weeks (**B**). DAPI was used for nuclear staining. Note that sorafenib decreased the expression of RAF1 in SP-derived tumors compared with the vehicle control after 4 weeks, which is consistent with the results shown in Fig. 5C. Scale bar, 200 μm.

In support of the immunoblot data on RAF1 (Fig. 5), immunofluorescence studies showed decreased expression of RAF1 in HHUA-SP-derived tumors after 2 and 4 weeks of sorafenib treatment compared with vehicle treatment (Fig.6A, B , respectively). Sorafenib decreased RAF1 expression in HHUA-MP-derived tumors after two weeks of treatment (Fig. 6A) but had little effect on RAF1 expression in HHUA-MP-derived tumors after 4 weeks of treatment (Fig. 6B). Immunofluorescence studies also revealed a similar tendency in ZEB1 expression as revealed by immunoblot data (Additional file 7: Fig. S7A and B). Taken together, these findings suggest that sorafenib may act as a CSC-selective drug by targeting signaling pathways associated with tumorigenesis, including RAF1-mediated cell proliferation and ZEB1-mediated EMT.

## Discussion

Both marker-dependent and marker-independent procedures are available for identification, isolation and characterization of CSCs [25]. We chose the SP method to isolate endometrial CSCs for the following reasons. First, SP methods have been successfully employed for identifying and isolating endometrial stem/progenitor cells from normal human endometrium [26, 27]. In particular, we demonstrated, using a cell tracking assay in combination with a human endometrial tissue reconstitution assay [28], that endometrial SP cells contribute to the generation of various lineages of endometrial cells including glandular, stromal, and endothelial cells [27]. Given that CSCs most likely arise from adult stem/progenitor cells [29] and that CSCs, therefore, may inherit some properties of adult stem/progenitor cells such as the SP phenotype, it is highly likely that the SP method would enable identification and isolation of authentic endometrial CSCs. Secondly, because cancer SP cells highly express three ATP-binding cassette (ABC) transporters, ABCB1 (also known as MDR1/P-glycoprotein), ABCC1, and ABCG2 (also known as BCRP) [30], they are thought to be chemo-resistant tumor-initiating cells [30]. Indeed, the two transporters ABCB1/MDR1 and ABCG/BCRP, which define the SP fraction, reduce the intracellular concentrations of various chemotherapeutic agents [30]. The objective of this study was to search for endometrial CSC-selective drugs to treat CSC-driven metastasis, recurrence, and chemoresistance. We, therefore, explored endometrial CSCs with both chemoresistance and tumor-initiating properties, rather than tumor-initiating properties alone, because such CSCs might be responsible for endometrial cancer associated with a greater clinical burden, including chemoresistance.

Among the endometrial cancer cell lines tested here, only HHUA cells contained a small but significant number of CSCs with an SP phenotype. This finding, however, did not exclude the possibility that the other endometrial cancer cell lines might have different types of CSCs without an SP phenotype. Indeed, CSCs with different phenotypes and markers may exist in the same histotype of cancer [4–6]. In addition to the heterogeneity of CSCs, even cancer cell lines can be rather heterogeneous [31, 32], which may account for the presence of both CSCs and the non-CSCs in HHUA cells.

It remains to be elucidated how CSCs (HHUA-SP cells) are involved in the malignant potential of HHUA cells, because HHUA cells originated from a well-differentiated endometrial adenocarcinoma that was likely treatment-sensitive and had a good prognosis compared to the poorly/undifferentiated carcinoma. The frequency of CSCs may depend on the type and stage of the tumor, i.e., poorly differentiated tumors may contain higher frequencies of CSCs [6]. Moreover, the precise nature of CSCs, i.e., the metastatic and chemoresistance potentials, may also differ between the well-differentiated and poorly/undifferentiated tumors.

To screen endometrial CSC-targeting drugs, a sufficient number of endometrial CSCs must be obtained. Several endometrial cancer cell lines, including Ishikawa and HEC1, contain SP cells that exhibit stem cell properties [33, 34]. Thus, we initially decided to use both cell lines to obtain adequate amounts of CSCs. However, we detected no or very few CSCs in either HEC1 (data not shown) or Ishikawa cells (Additional file 1: Fig. S1). Thus, we searched for other endometrial cell lines better suited for these experiments. Among the various types of endometrium-derived cells evaluated, we found that only the HHUA endometrial cancer cell line stably and consistently contained a reserpine-sensitive SP fraction with stem cell properties. Those characteristics included high expression of MDR1; high capacities for proliferation, colony formation, and invasion; and in vivo generation of tumors overexpressing the proliferation-associated markers Ki67, c-MYC, and pERK. Those properties were absent in the non-SP fraction (Fig. 5B, C). pERK plays critical roles in cell cycle progression via c-MYC stabilization, EMT via ZEB1 induction, apoptosis, autocrine cytokine loops, cancer metabolism, and protein translation [35, 36]. Consistently, pERK may drive c-MYC and Ki67 expression, resulting in the formation of larger tumors from HHUA-SP cells than from non-SP HHUA cells (Fig. 5B, C ). Also, there was a tendency for ZEB1 to be upregulated in HHUA-SP-derived tumors compared with non-SP HHUA-derived tumors, which was probably due to the increased pERK level (Fig. 5B, C). CTNNB1, a known endometrial cancer stem cell marker [21], was upregulated in HHUA-SP-derived tumors compared to non-SP HHUA-derived tumors (Fig. 1G). Thus, we obtained solid evidence for the cancer stemness of HHUA-SP cells, thereby providing us with HHUA cells to explore endometrial CSC-targeting drugs.

Traditional drug discovery and development consist of multiple processes that are time- and cost-consuming, laborious, and highly risky. Recently, drug repositioning, also known as drug repurposing, has emerged as an effective strategy to determine new indications for existing drugs, an approach that decreases time, cost, labor and risk [37]. In this study, we focused on previously approved drugs that could target CSC-specific or -associated signaling pathways. The first drug type evaluated was inhibitors of Wnt signaling because the Wnt pathway plays critical roles in the growth, differentiation, and function of various types of stem cells [38], including embryonic stem cells, adult stem cells, benign tumor stem cells [39] and CSCs. Thus, aberrant Wnt signaling provokes the initiation and expansion of tumors derived from CSCs [38]. The second drug type evaluated included inhibitors of angiogenesis. Angiogenesis and blood supply facilitate cancer progression, recurrence, and metastasis [40]. In particular, vascular niches maintain the stem cell phenotype and activities of CSCs as well as normal stem cells [40]. Furthermore, CSCs are capable of transdifferentiating into endothelial-like cells, thereby enhancing neovascularization, referred to as vasculogenic mimicry [41]. Indeed, putative endometrial stem/progenitor cells preferentially express typical endothelial markers including CD31 and vascular endothelial growth factor receptor and have in vivo potential for angiovasculogenesis [26, 27]. Given that endometrial CSCs may exhibit properties similar to those of endometrial stem cells [42], the same as other types of CSCs [29, 43], it is possible that targeting angiovasculogenesis has a therapeutic impact not only on endometrial CSC niches but also on endometrial CSCs themselves. The third class of candidate drugs targeting endometrial CSCs were those inhibiting the EMT. The EMT process is closely involved in the generation, expansion, invasion, and metastasis of CSCs and ultimately contributes to cancer progression and chemoresistance [44]. We searched for drugs that exert a selective inhibitory effect on CSCs irrespective of their effect on non-stem cancer cells. We demonstrated that sorafenib, an antiangiogenic agent, exhibits the greatest CSC-selective inhibitory effects on the proliferation, survival, invasion, and in vivo tumorigenesis of HHUA cells among the three categories of drugs evaluated. We thus selected sorafenib as a CSC-targeting drug for further analyses.

Sorafenib is an oral multi-kinase inhibitor with anticancer activity against a wide variety of cancers and is currently approved for the treatment of patients with advanced unresectable hepatocellular carcinoma, advanced renal cell carcinoma, or progressive, locally advanced, or metastatic differentiated thyroid carcinoma [45]. Sorafenib inhibits mainly RAFs, such as RAF1 involved in the MAPK/ERK pathway, and also attenuates several tyrosine kinases, thereby suppressing tumor cell and vessel growth and growth factor production [45, 46].

Consistent with this, treatment with sorafenib, a RAF/ERK inhibitor, significantly decreased tumor weight and expression of RAF1 and ZEB1, a downstream effector of the RAF/ERK pathway, in HHUA-SP-derived tumors but not non-SP-derived tumors (Figs. 4–6). In addition, the levels of pERK and its downstream effector, c-MYC, tended to decrease after treatment with sorafenib in HHUA-SP-derived tumors, but not non-SP-derived tumors (Fig. 5B, C). Taken together with the higher levels of pERK and c-MYC in HHUA-SP-derived compared with HHUA-MP-derived tumors (Fig. 5C), these results suggest that the RAF/ERK pathway may play a greater role in HHUA-SP- than non-SP-derived tumors, rendering the former to be more susceptible to sorafenib, a representative RAF/ERK inhibitor. The upstream kinase governing the RAF/ERK pathway is RAS [35, 36], which is constitutively active via its gene mutation (K-RasV12) in HHUA cells [47]. It is tempting to speculate that HHUA-SP cells may acquire stronger downstream RAS signaling activity compared with HHUA-MP cells. In support of this, the RAS/RAF/MEK/ERK pathway has been shown to have roles in CSCs, senescence, aging, and sensitivity to targeted therapies [48]. These findings collectively raise the possibility that sorafenib is an effective treatment for endometrial cancer with RAS mutations or upregulated RAF/ERK pathway activity, as suggested previously [49].

Based on the prominent role of the RAS/RAF/MEK/ERK pathway in angiogenesis and progression of endometrial cancer [50, 51], a phase II study of sorafenib treatment for advanced endometrial cancer was conducted; however, the results indicated very modest effects only [52]. To elucidate the mechanism of this resistance and introduce a new approach to improve sorafenib efficacy in patients with endometrial cancer, Eritja et al. identified sorafenib-induced activation of protective autophagic responses as a mechanism underlying the resistance to sorafenib [53]. Using primary endometrial cancer orthoxenografts, those authors also demonstrated, that targeting autophagy enhances sorafenib cytotoxicity, thereby suppressing tumor growth and metastasis [53]. Here, we showed that sorafenib selectively acted on endometrial CSCs, i.e., those with upregulated RAF/ERK pathway activity rather than all endometrial cancer cells. This finding explains why sorafenib benefits some patient populations without producing a dramatically increased response rate, as revealed in a phase II clinical trial [52]. It is possible that selective inhibition of endometrial CSCs by sorafenib may not result in an apparent reduction in tumor size but may contribute to changes in tumor characteristics such as invasiveness and aggressiveness through targeting CSCs within the tumor. One possible clinical application is suggested by our results. That is, we could first examine whether MDR-positive cells, i.e. SP cells, are present in the surgical or biopsy specimens of endometrial cancer. If they were present, we should consider the use of sorafenib, particularly for treatment of advanced or recurrent endometrial cancer.

## Conclusion

This is the first report to demonstrate that HHUA cells contain stable and abundant SP cells that exhibit CSC properties, including high MDR1 expression and strong capabilities of cell proliferation, colony formation, invasion, and in vivo generation of tumors overexpressing the proliferation-associated markers Ki67, c-MYC, and pERK, compared with non-SP cells. Based on the cancer stemness of HHUA-SP cells, we tested the effects of 14 putative CSC-targeting drugs on HHUA-SP and non-SP HHUA cell functions, including proliferation, invasion, and/or in vivo tumor formation. Among the drugs tested, sorafenib was eventually identified as an endometrial CSC-selective drug in that it inhibited SP cell functions specifically by targeting the RAF/ERK pathway. Our strategy in combination with the use of HHUA cells will be useful for identifying endometrial CSC-selective drugs.

## Supplementary Information

**Additional file 1. Figure S1**: Flow cytometric distribution of the side population (SP) and main population (MP) of various cell lines. The cells were stained with Hoechst 33342 in the absence (R–) or presence (R+) of reserpine. Note that only HHUA cells contained a reserpine-sensitive SP fraction. The data derived from HEC1 are not shown, but HEC1 had no or very few SP cells.

**Additional file 2. Figure S2**: Flow cytometric analysis of apoptosis in HHUA cells treated with or without niclosamide or sorafenib for 7 days. Upper three panels: representative FSC (forward scatter) vs. SSC (side scatter) dot plots of HHUA cells treated as indicated. Lower three panels: representative flow cytometry plots using Annexin V-FITC/PI staining for the presence of apoptotic HHUA cells treated as indicated. The percentages of FITC(+)/ PI(+) cells (late apoptotic and necrotic cells) and FITC(+)/PI(-) cells (early apoptotic cells) are shown as the mean ± SEM from three independent samples.

**Additional file 3. Figure S3**: Magnified images of MDR1 and Ki67 immunostaining of HHUA-SP- or -MP-derived tumor 2 weeks after treatment. Immunofluorescence staining of MDR1 (A) and Ki67 (B) in HHUA-SP- and -MP-derived tumors in mice treated orally with or without sorafenib for 2 weeks (A, B). Small red boxes mark regions shown at higher magnification in the adjacent panels as indicated by the corresponding number. Scale bar, 200 μm (yellow) and 25 μm (white).

**Additional file 4. Figure S4**: Macroscopic images, tumor volumes, and magnified MDR1 and Ki67 immunostaining images of HHUA-SP or -MP-derived tumor after 4 weeks of treatment. A. Volume and gross appearance of tumors derived from HHUA-SP and -MP cells after 4 weeks of treatment without and with sorafenib. Each dot in the right-handed graph indicates the tumor volume of an individual mouse. The tumor volume was calculated based on the tumor diameter. Scale bars, 1 mm. B, C. Immunofluorescence staining of MDR1 (B) and Ki67 (C) in HHUA-SP- and -MP-derived tumors in mice treated orally with or without sorafenib for 4 weeks. Small red boxes mark regions shown at higher magnification in the adjacent panels as indicated by the corresponding number. Scale bar, 200 μm (yellow) and 25 μm (white).

**Additional file 5. Figure S5**: Effects of sorafenib on BCL2 expression in HHUA-SP- and -MP-derived tumors in mice. Immunofluorescence staining of BCL2 and VM in HHUA-SP- and -MP-derived tumors in mice treated orally with or without sorafenib for 4 weeks. DAPI was used for nuclear staining. Small boxes delineate regions shown at higher magnification in the adjacent panel as indicated. Scale bar, 200 μm (white) and 25 μm (Red).

**Additional file 6. Figure S6**:Effects of sorafenib on the phosphorylation status of ERK and AKT in HHUA-SP- and -MP-derived tumors in mice. Each bar indicates the mean ± SEM relative density of each indicated phosphorylated protein obtained from three individual HHUA-SP- and -MP-derived tumor samples, as described in Figure 5B. The protein levels were normalized to that of total protein. *, P < 0.05.

**Additional file 7. Figure S7**: Effects of sorafenib on ZEB1 expression in HHUA-SP- and -MP-derived tumors in mice A. Immunofluorescence staining of ZEB1 and VM in HHUA-SP- and -MP-derived tumors in mice treated orally with or without sorafenib for 4 weeks. DAPI was used for nuclear staining. Scale bar, 200 μm (white). B. Each bar indicates the mean ± SEM relative density of each indicated ZEB1-positive cell obtained from 5 random photos of HHUA-SP- and -MP-derived tumor samples, as described in (A). The percentages of ZEB1-positive cells were calculated as the ratio of ZEB1 positive cells to VM positive cells.

## Data Availability

All data needed to evaluate the conclusions in the paper are present in the paper.

## Abbreviations

CSC: Cancer stem cells
SP: Side population
MP: Main population
MDR1: Multidrug resistance 1
ABC: ATP-binding cassette
EMT: Epithelial-to-mesenchymal transition
