# Supplementary figures and images for: Sorafenib targets and inhibits the oncogenic properties of endometrial cancer stem cells via the RAF/ERK pathway
Source: Stem Cell Res Ther. 2022 Jun 3;13:225. doi: 10.1186/s13287-022-02888-y (PMC9166406; doi:10.1186/s13287-022-02888-y)

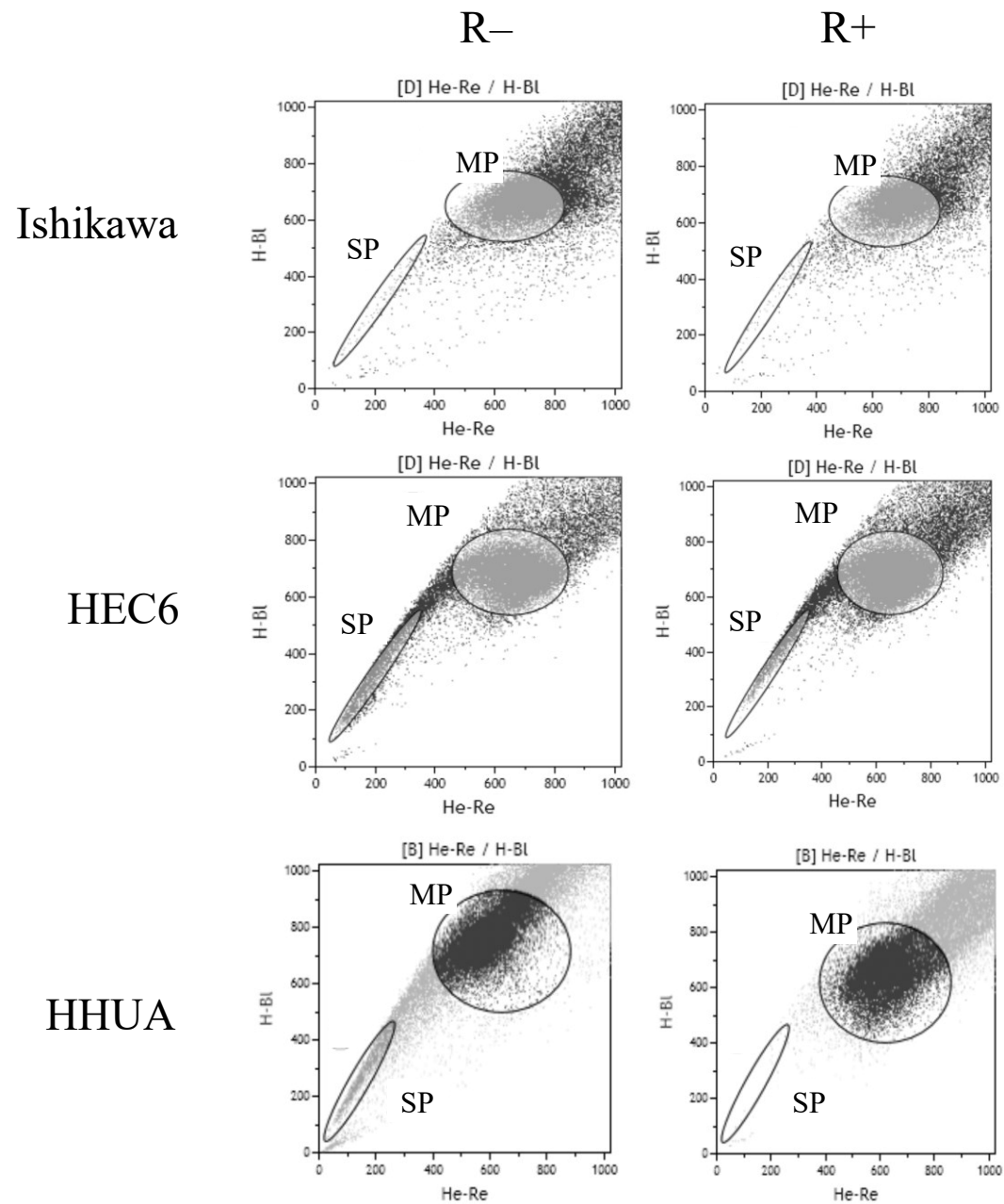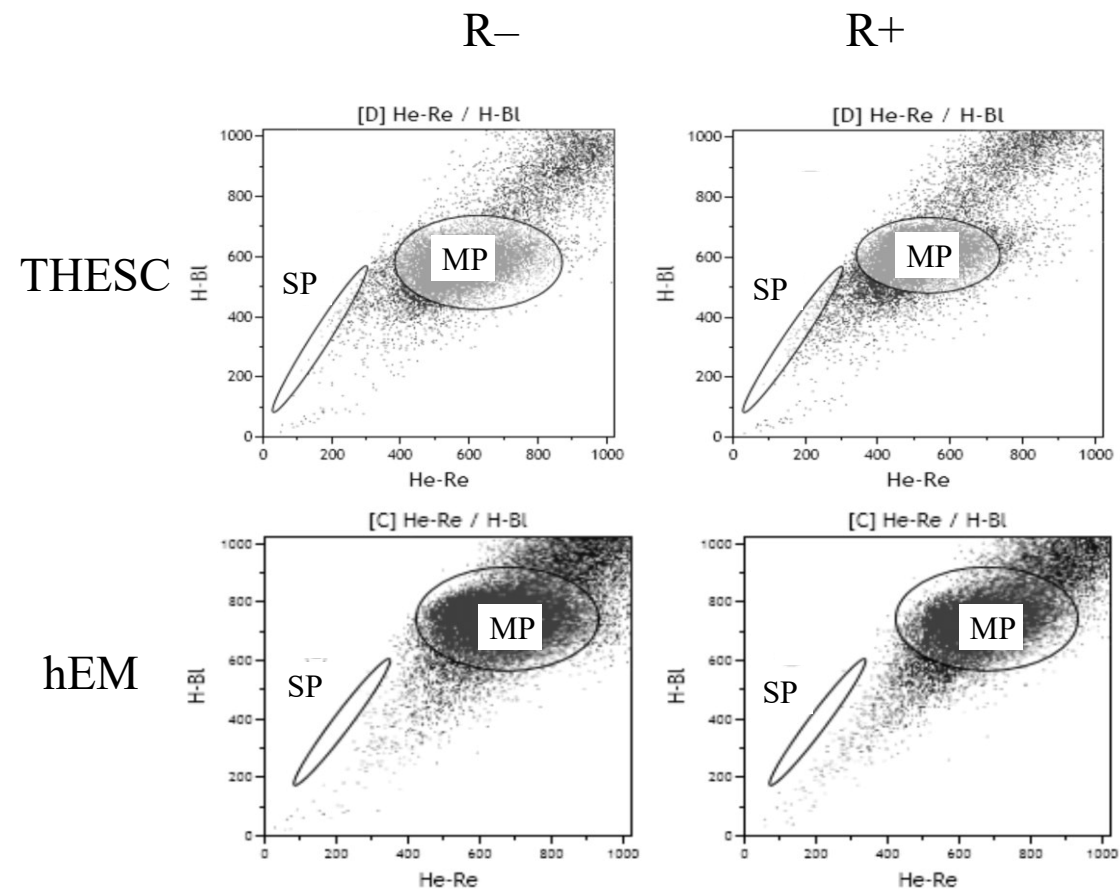

Supplementary Figure 1. Takao et al.

Supplement: Supplementary file 1 — Additional file 1. Figure S1: Flow cytometric distribution of the side population (SP) and main population (MP) of various cell lines. The cells were stained with Hoechst 33342 in the absence (R–) or presence (R+) of reserpine. Note that only HHUA cells contained a reserpine-sensitive SP fraction. The data derived from HEC1 are not shown, but HEC1 had no or very few SP cells. [file 13287_2022_2888_MOESM1_ESM.pdf]

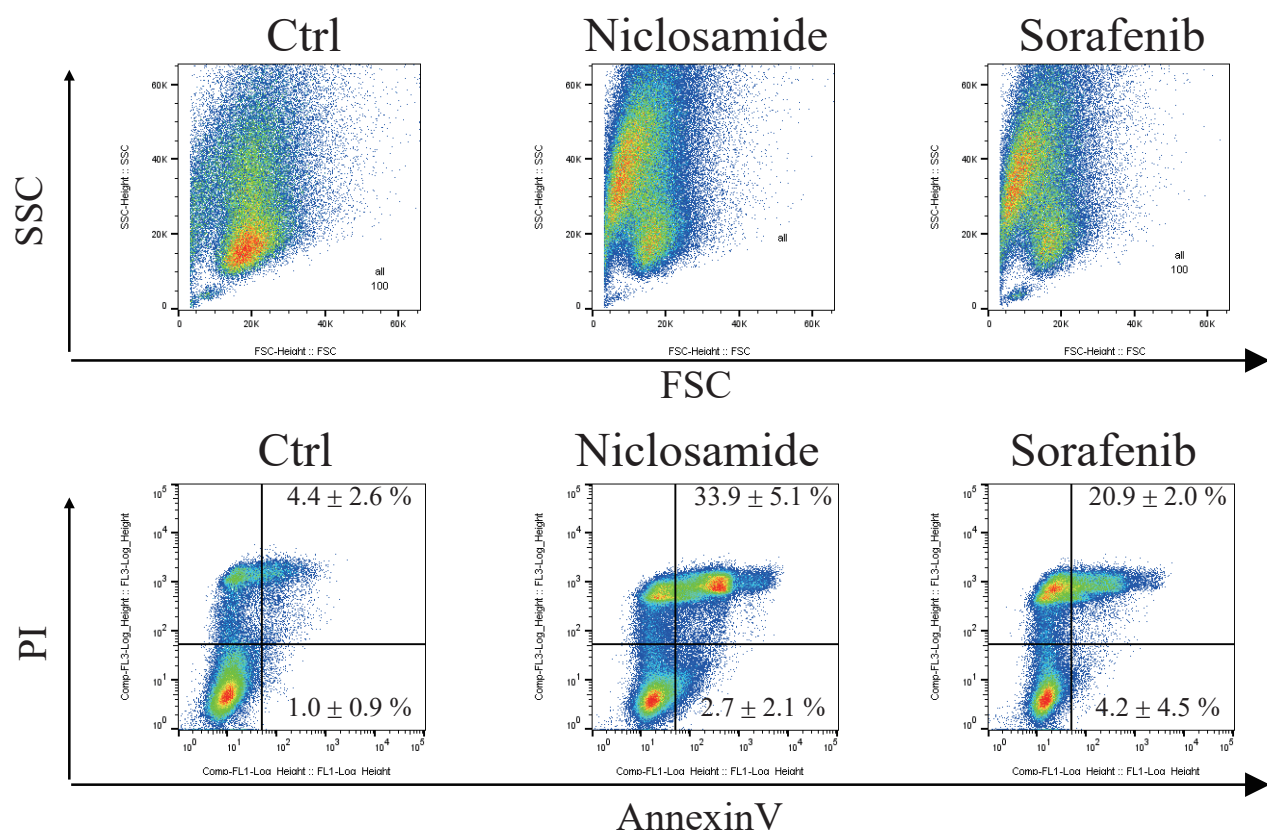

Supplementary Figure 2. Takao et al.

Supplement: Supplementary file 2 — Additional file 2. Figure S2: Flow cytometric analysis of apoptosis in HHUA cells treated with or without niclosamide or sorafenib for 7 days. Upper three panels: representative FSC (forward scatter) vs. SSC (side scatter) dot plots of HHUA cells treated as indicated. Lower three panels: representative flow cytometry plots using Annexin V-FITC/PI staining for the presence of apoptotic HHUA cells treated as indicated. The percentages of FITC(+)/ PI(+) cells (late apoptotic and necrotic cells) and FITC(+)/PI(-) cells (early apoptotic cells) are shown as the mean ± SEM from three independent samples. [file 13287_2022_2888_MOESM2_ESM.pdf]

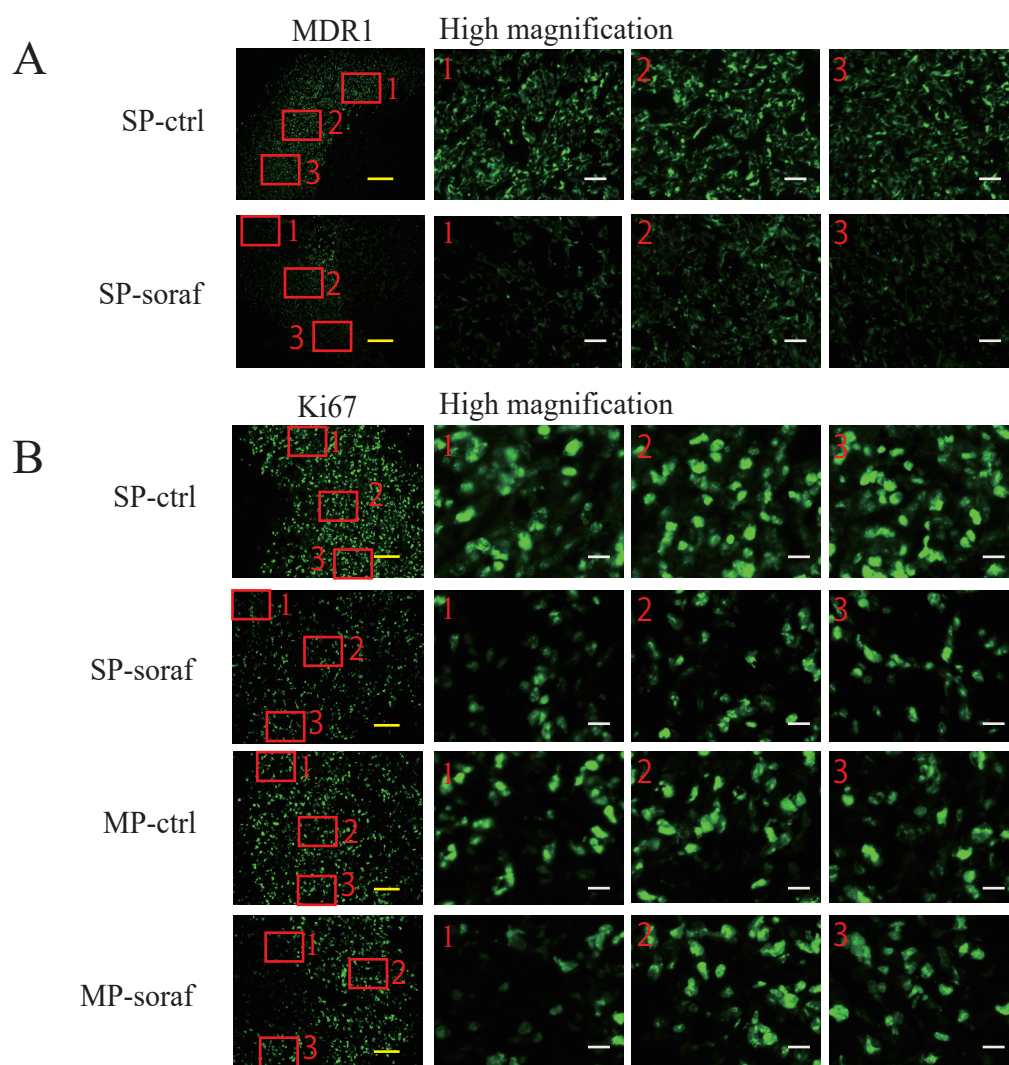

Supplementary Figure 3. Takao et al.

Supplement: Supplementary file 3 — Additional file 3. Figure S3: Magnified images of MDR1 and Ki67 immunostaining of HHUA-SP- or -MP-derived tumor 2 weeks after treatment. Immunofluorescence staining of MDR1 (A) and Ki67 (B) in HHUA-SP- and -MP-derived tumors in mice treated orally with or without sorafenib for 2 weeks (A, B). Small red boxes mark regions shown at higher magnification in the adjacent panels as indicated by the corresponding number. Scale bar, 200 μm (yellow) and 25 μm (white). [file 13287_2022_2888_MOESM3_ESM.pdf]

A

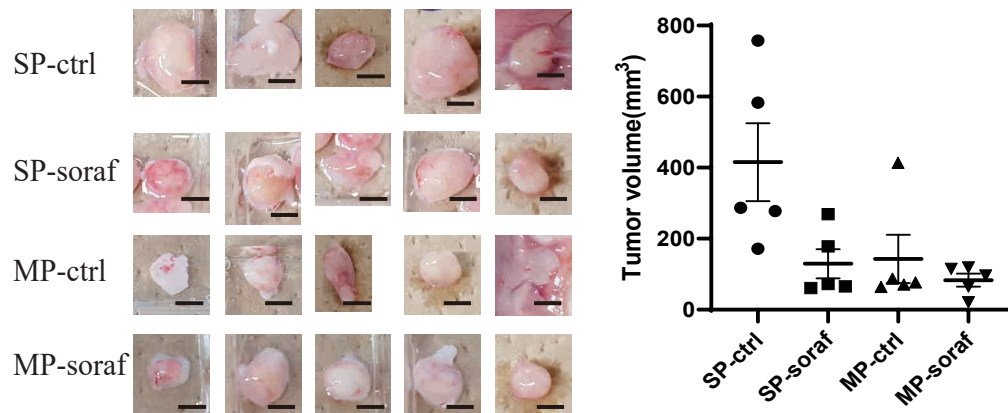

B

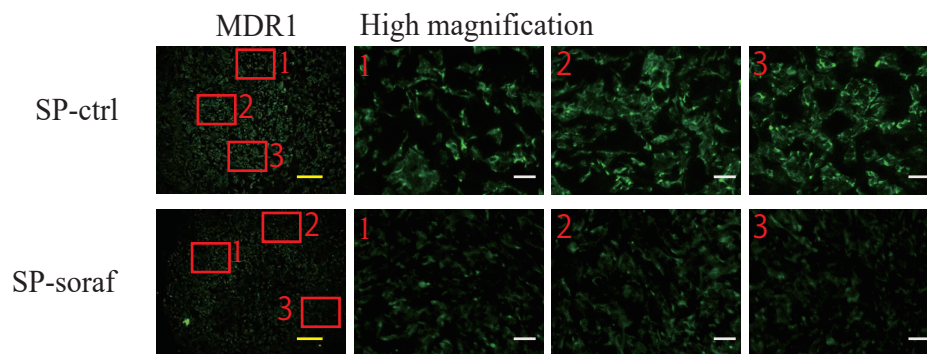

C

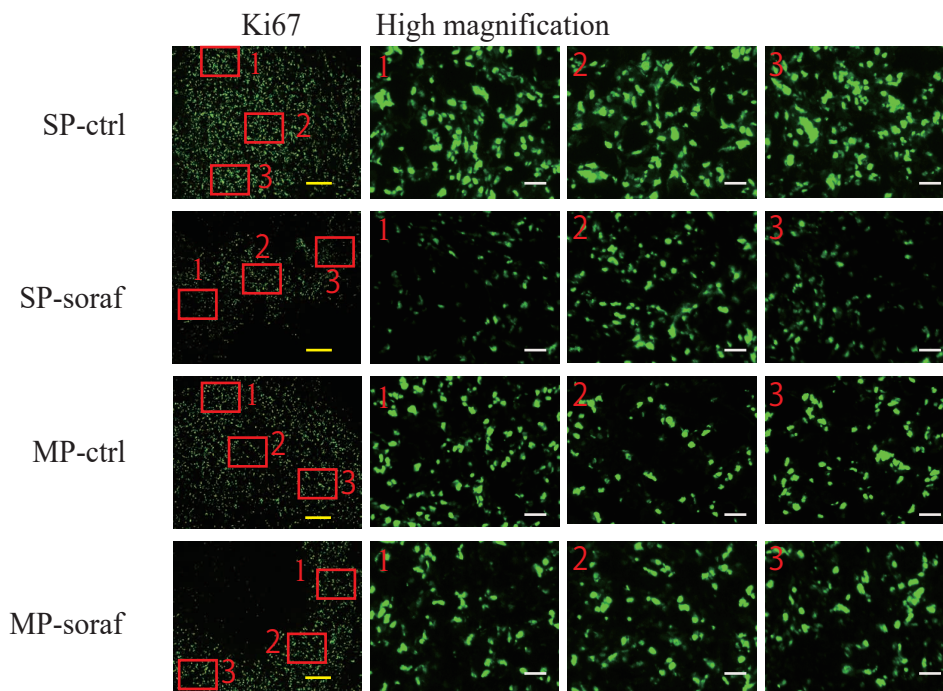

Supplementary Figure 4. Takao et al.

Supplement: Supplementary file 4 — Additional file 4. Figure S4: Macroscopic images, tumor volumes, and magnified MDR1 and Ki67 immunostaining images of HHUA-SP or -MP-derived tumor after 4 weeks of treatment. A. Volume and gross appearance of tumors derived from HHUA-SP and -MP cells after 4 weeks of treatment without and with sorafenib. Each dot in the right-handed graph indicates the tumor volume of an individual mouse. The tumor volume was calculated based on the tumor diameter. Scale bars, 1 mm. B, C. Immunofluorescence staining of MDR1 (B) and Ki67 (C) in HHUA-SP- and -MP-derived tumors in mice treated orally with or without sorafenib for 4 weeks. Small red boxes mark regions shown at higher magnification in the adjacent panels as indicated by the corresponding number. Scale bar, 200 μm (yellow) and 25 μm (white). [file 13287_2022_2888_MOESM4_ESM.pdf]

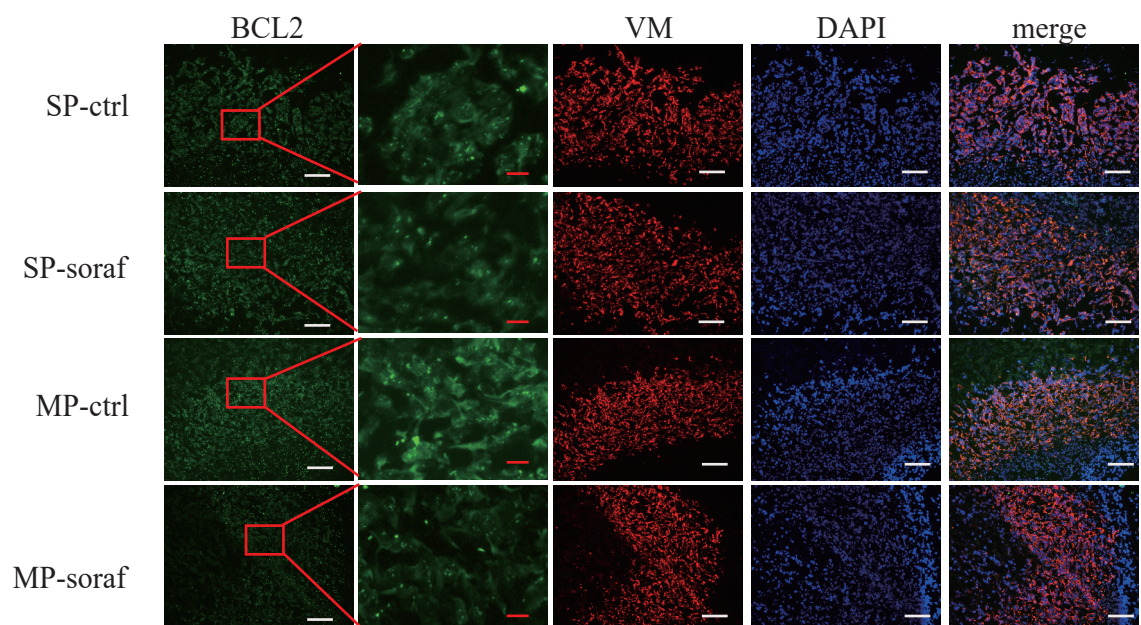

Supplementary Figure 5. Takao et al.

Supplement: Supplementary file 5 — Additional file 5. Figure S5: Effects of sorafenib on BCL2 expression in HHUA-SP- and -MP-derived tumors in mice. Immunofluorescence staining of BCL2 and VM in HHUA-SP- and -MP-derived tumors in mice treated orally with or without sorafenib for 4 weeks. DAPI was used for nuclear staining. Small boxes delineate regions shown at higher magnification in the adjacent panel as indicated. Scale bar, 200 μm (white) and 25 μm (Red). [file 13287_2022_2888_MOESM5_ESM.pdf]

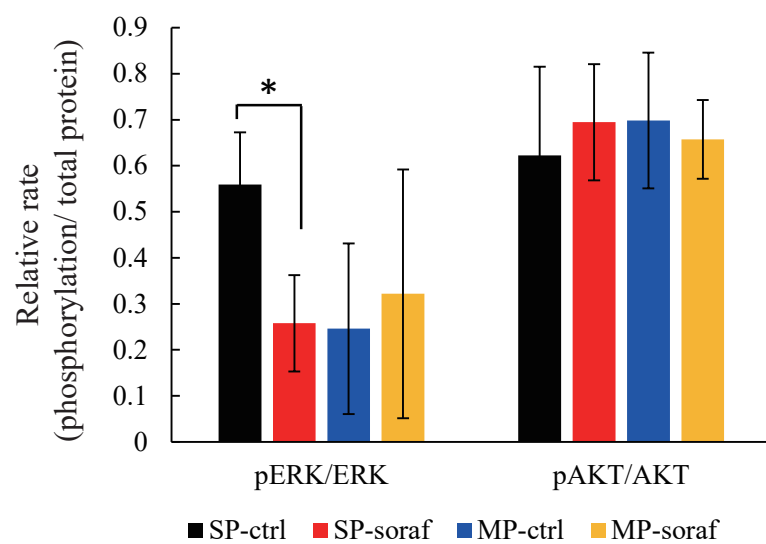

Supplementary Figure 6. Takao, et al.

Supplement: Supplementary file 6 — Additional file 6. Figure S6:Effects of sorafenib on the phosphorylation status of ERK and AKT in HHUA-SP- and -MP-derived tumors in mice. Each bar indicates the mean ± SEM relative density of each indicated phosphorylated protein obtained from three individual HHUA-SP- and -MP-derived tumor samples, as described in Figure 5B. The protein levels were normalized to that of total protein. *, P < 0.05. [file 13287_2022_2888_MOESM6_ESM.pdf]

A

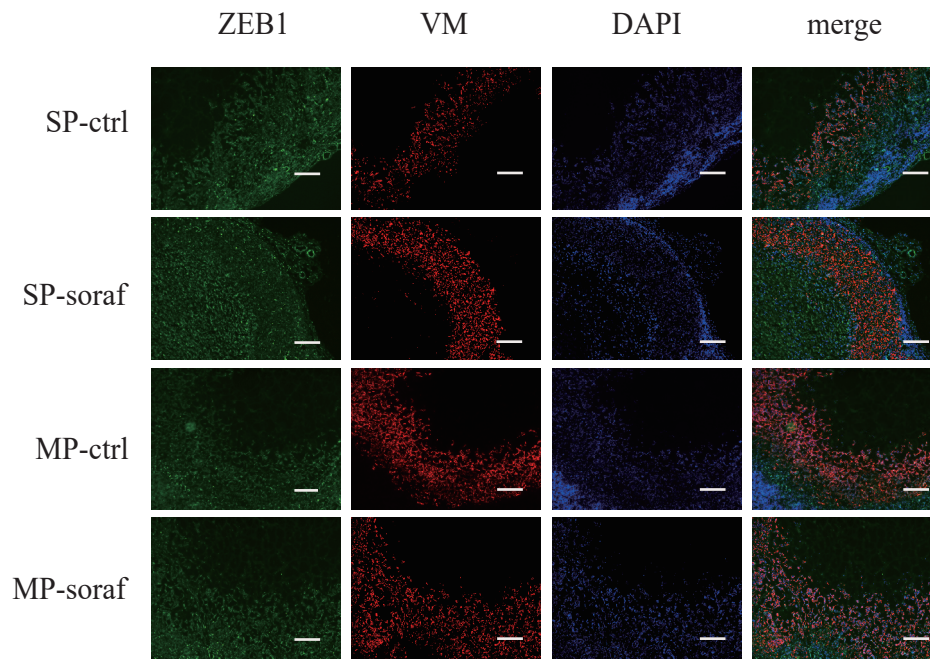

B

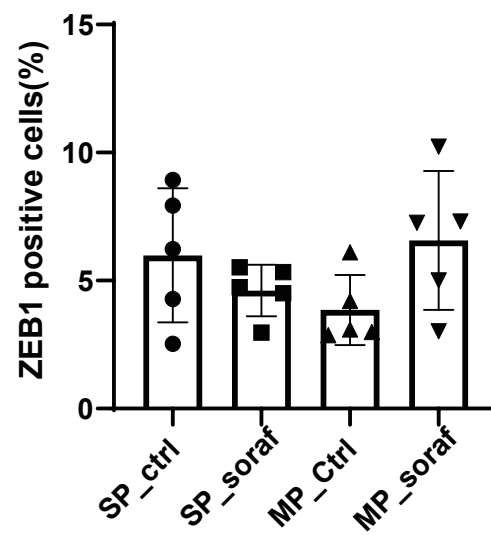

Supplementary Figure 7. Takao et al.

Supplement: Supplementary file 7 — Additional file 7. Figure S7: Effects of sorafenib on ZEB1 expression in HHUA-SP- and -MP-derived tumors in mice A. Immunofluorescence staining of ZEB1 and VM in HHUA-SP- and -MP-derived tumors in mice treated orally with or without sorafenib for 4 weeks. DAPI was used for nuclear staining. Scale bar, 200 μm (white). B. Each bar indicates the mean ± SEM relative density of each indicated ZEB1-positive cell obtained from 5 random photos of HHUA-SP- and -MP-derived tumor samples, as described in (A). The percentages of ZEB1-positive cells were calculated as the ratio of ZEB1 positive cells to VM positive cells. [file 13287_2022_2888_MOESM7_ESM.pdf]
